# Pu-Erh Tea Extract Induces the Degradation of FET Family Proteins Involved in the Pathogenesis of Amyotrophic Lateral Sclerosis

**DOI:** 10.1155/2014/254680

**Published:** 2014-04-07

**Authors:** Yang Yu, Shuhei Hayashi, Xianbin Cai, Chongye Fang, Wei Shi, Hiroko Tsutsui, Jun Sheng

**Affiliations:** ^1^Key Laboratory for Molecular Enzymology and Engineering, The Ministry of Education, Jilin University, Changchun 130012, China; ^2^Department of Pu-Erh Tea and Medical Science, Hyogo College of Medicine, 1-1 Mukogawa-cho, Nishinomiya, Hyogo 663-8501, Japan; ^3^State Key Laboratory of Phytochemistry and Plant Resources in West China, Kunming Institute of Botany, Chinese Academy of Sciences, Kunming 650201, China; ^4^Department of Microbiology, Hyogo College of Medicine, 1-1 Mukogawa-cho, Nishinomiya, Hyogo 663-8501, Japan; ^5^Key Laboratory of Pu-Erh Tea Science, The Ministry of Education, Yunnan Agricultural University, Kunming 650201, China

## Abstract

FET family proteins consist of fused in sarcoma/translocated in liposarcoma (FUS/TLS), Ewing's sarcoma (EWS), and TATA-binding protein-associated factor 15 (TAF15). Mutations in the copper/zinc superoxide dismutase (SOD1), TAR DNA-binding protein 43 (TDP-43), and FET family proteins are associated with the development of amyotrophic lateral sclerosis (ALS), a fatal neurodegenerative disease. There is currently no cure for this disease and few effective treatments are available. Epidemiological studies indicate that the consumption of tea is associated with a reduced risk of developing neurodegenerative diseases. The results of this study revealed that components of a pu-erh tea extract (PTE) interacted with FET family proteins but not with TDP-43 or SOD1. PTE induced the degradation of FET family proteins but had no effects on TDP-43 or SOD1. The most frequently occurring ALS-linked FUS/TLS mutant protein, R521C FUS/TLS, was also degraded in the presence of PTE. Furthermore, ammonium chloride, a lysosome inhibitor, but not lactacystin, a proteasome inhibitor, reduced the degradation of FUS/TLS protein by PTE. PTE significantly reduced the incorporation of R521C FUS/TLS into stress granules under stress conditions. These findings suggest that PTE may have beneficial health effects, including preventing the onset of FET family protein-associated neurodegenerative diseases and delaying the progression of ALS by inhibiting the cytoplasmic aggregation of FET family proteins.

## 1. Introduction

Amyotrophic lateral sclerosis (ALS) is one of the major motor neuron diseases. It is a rapidly progressive neurological disorder that involves the degeneration of motor neurons, leading to paralysis and death [1]. In most cases, the disease develops in subjects aged between 40 and 60 years. Currently, there is no effective treatment available for preventing the inexorable neurodegeneration that eventually results in death within 1–5 years after the symptoms emerge.

Most cases of ALS are sporadic, but approximately 10% are familial. Several mutant genes have been identified in patients with familial ALS. The first mutations identified were in the* SOD1* gene on chromosome 21 [2]. Recently, TDP-43 and FET family proteins have also been identified as being involved in the development of both ALS and frontotemporal lobar degeneration (FTLD) [3–9]. FET family proteins and TDP-43 are RNA-binding proteins that are structurally and functionally similar [10, 11], which have been associated with multiple nuclear and cytoplasmic steps of RNA processing [12]. The accumulation of FUS/TLS in the cytoplasm of the nervous systems of patients with FUS/TLS mutations disrupts its normal nuclear localization [13]. Most of the FUS/TLS mutations cluster in the C-terminus of the FUS/TLS protein, while R521C FUS/TLS is the most common mutation of FUS/TLS-associated ALS [14]. Although the mechanisms responsible for the aggregation of TDP-43 and FET family proteins are currently unknown, the increased stability of the mutant or wild-type TDP-43 protein has the potential for causing toxicity through abnormal proteostasis and RNA dysregulation [15]. A histone acetyltransferase inhibitor, referred to as anacardic acid, was reported to rescue the abnormal ALS motor neuron phenotype through the inhibition of TDP-43 protein expression [16]. Transgenic mice that overexpress human FUS/TLS have limb paralysis and death occurs by 12 weeks in homozygous mice [17]. The overexpression of human mutant FUS/TLS (R521C substitution) leads to progressive paralysis that resembles ALS in transgenic rats [18].

Pu-erh tea is mainly produced in the Yunnan Province of China and is widely consumed in southeastern Asia owing to its unique flavor and potential health benefits. Pu-erh tea, unlike green tea, is a type of fermented tea and includes microbial metabolites. A number of* in vitro* and animal studies have demonstrated that pu-erh tea has antioxidant [19] and antiobesity properties [20]. Pu-erh tea also has strong anticancer protective effects [21] and can ameliorate diabetic nephropathy [22]. Several compounds are produced during the postfermentation of pu-erh tea. These compounds are produced from the degradation of proteins and carbohydrates and the oxidation of polyphenols by the enzymatic action of microorganisms [23]. The chemical characteristics and bioactivities of pu-erh tea remain unclear.

The identification of proteins that bind to components of pu-erh tea extract (PTE) will aid in our understanding of the molecular and biochemical mechanisms that underlie its effects. Moreover, the identification of the target proteins that associate with PTE will be useful in the development of new strategies with the objective of understanding its biological functions of pu-erh tea. In this study, we used PTE Sepharose 6B beads and MALDI-TOF MS to purify and identify proteins associated with PTE from cell lysates. The results of this study revealed that PTE interacts with FET family proteins and induces the degradation of FET family proteins. The findings suggest that pu-erh tea is a potential natural source of protection against neurodegenerative diseases that are associated with FET family proteins.

## 2. Materials and Methods

### 2.1. Reagents and Antibodies

PTE, green tea extract (GTE), and black tea extract (BTE) were kindly supplied by the China Academy of Pu-Erh Tea Research [21, 22]. These extracts were dissolved in distilled water and adjusted to pH 7.4 with 1 M NaOH. Ammonium chloride (NH_4_Cl), a lysosomal inhibitor, was purchased from Wako Pure Chemical Industries (Osaka, Japan). Lactacystin (Calbiochem, Bad Soden, Germany) was dissolved in dimethyl sulfoxide (DMSO). Sodium arsenite solution was purchased from EMD Millipore (Darmstadt, Germany). Anti-FUS/TLS, anti-LDLR (low density lipoprotein receptor), anti-TDP-43, anti-V5, anti-*β*-actin, and horseradish peroxidase-conjugated secondary antibody were purchased from Santa Cruz Biotechnology (Santa Cruz, CA), Abnova (Taipei City, Taiwan), Cell Signaling Technology (Beverley, MA), Invitrogen (Carlsbad, CA), Sigma (St. Louis, MO), and Thermo Scientific (Waltham, MA), respectively. Anti-EWS and anti-TAF15 were purchased from Cell Signaling Technology (Beverley, MA). The SOD1 antibody was a kind gift from Dr. Noriko Fujiwara.

### 2.2. Cell Culture

The human neuroblastoma cell line (SK-N-SH) was purchased from the European Collection of Cell Culture (Salisbury, UK). SK-N-SH cells and HEK 293T cells were cultured in DMEM supplemented with 10% fetal bovine serum. The human acute T lymphoblastic leukemia cell line (MOLT-3) was grown in RPMI-1640 medium supplemented with 10% fetal bovine serum.

### 2.3. Preparation of Tea Extract Sepharose 6B Beads

Epoxy-activated Sepharose 6B beads were purchased from GE Healthcare Bio-Sciences Corporation (Little Chalfont, UK). The equal amounts of PTE, GTE, and BTE were prepared according to the same procedure. The Sepharose 6B beads that served as a control were prepared according to the same procedure.

### 2.4. Purification of PTE-Associated Protein

Briefly, 1 × 10^7^ MOLT-3 cells were lysed in buffer (1% Triton X-100, 10 mM Tris, 150 mM NaCl, pH 7.6). A four-step procedure for the purification of the PTE-associated proteins was performed. In the first step, the lysate was added to Sepharose 6B beads and incubated with gentle rocking overnight at 4°C. The samples were then centrifuged and the supernatant was collected. In the second step, the supernatant was added to the GTE Sepharose 6B beads and incubated with gentle rocking overnight at 4°C, and the supernatant was collected by centrifugation. In the third step, the second-step supernatant was added to BTE Sepharose 6B beads, which were then incubated overnight at 4°C, and the supernatant was isolated by centrifugation. In the final step, the third-step supernatant was added to PTE Sepharose 6B beads and the supernatant was incubated overnight at 4°C. The PTE Sepharose 6B beads were then washed five times with lysis buffer. The proteins that were bound to the beads were analyzed on SDS-PAGE. Cell lysates without any treatment served as the controls. The gels were stained with Coomassie brilliant blue (Nacalai, Kyoto, Japan).

### 2.5. MALDI-TOF MS Analysis

The protein band of interest was excised from the gel and analyzed by matrix-assisted laser desorption ionization-time-of-flight mass spectrometry (MALDI-TOF MS) (Microflex LRF 20, Bruker Daltonik GmbH, Bremen, Germany). The peptide masses were entered into Mascot (http://www.matrixscience.com/), and the NCBI database was searched to identify the protein.

### 2.6. Pull-Down Assay

Cells lysates were obtained with lysis buffer (1% Triton X-100, 10 mM Tris, 150 mM NaCl, pH 7.6) and incubated with PTE Sepharose 6B beads (or Sepharose 6B beads as the control). After incubation with gentle rocking overnight at 4°C, the beads were washed five times with lysis buffer, and proteins that were bound to the beads were analyzed by Western blot.

### 2.7. Western Blot

Whole cell extracts were prepared with RIPA buffer (1% NP-40, 0.5% sodium deoxycholate, 1 mM EDTA, 50 mM Tris-Cl [pH 7.4], 150 mM NaCl, 25 mM NaF, 1 mM DTT, 1 mM Na_3_VO_4_, and 1 mM phenylmethylsulfonyl fluoride [PMSF]). Protein concentrations were determined with a BCA Protein Assay Kit (Pierce, Rockford, IL). The proteins were separated by SDS-PAGE, transferred to Immun-Blot PVDF membranes (Bio-Rad, USA) and blocked with 5% skimmed milk. The processed membranes were then incubated overnight at 4°C with primary antibodies. Horseradish peroxidase-conjugated secondary antibodies were then added and the membranes were incubated for 1 h at room temperature. The signal was detected with SuperSignal Dura Substrate (Pierce, Rockford, IL).

### 2.8. Cell Viability

Cell viability was quantified using Trypan blue stain (0.4%) and a Countess Automated Cell Counter (Invitrogen, Carlsbad, CA).

### 2.9. Plasmid Construction

The open reading frames of human FUS/TLS, FUS/TLS R521C mutant and LDLR were amplified from a human cDNA library and cloned into pcDNA3.1/V5/His-TOPO (Invitrogen, Carlsbad, CA) and then verified by sequencing. Primer sequences are shown in Table 1.

### 2.10. Transfection

A total of 1 × 10^6^ SK-N-SH cells were transfected with 2 *μ*g of FUS/TLS, FUS/TLS R521C mutant and LDLR plasmids using the Amaxa-Nucleofector-System (Lonza, Allendale, NJ, USA) according to the manufacturer's instructions. At 48 h after transfection, cells were treated with or without PTE (200 *μ*g/mL) for an additional 24 h and the cells were then collected for Western blot analysis. Transfections of HEK 293T cells were performed using X-tremeGENE HP (Roche, Mannheim, Germany) following the manufacturer's protocol.

### 2.11. Establishment of SK-N-SH Cell Lines Expressing R521C FUS/TLS

To generate R521C FUS/TLS-expressing cells, SK-N-SH cells were transfected by the Amaxa-Nucleofector-System. Transfected cells were cultured for 4–6 weeks in G418 (0.6 mg/mL). G418-resistant cells were screened for R521C FUS/TLS expression by Western blot.

### 2.12. RT-PCR

The expression of FUS/TLS mRNA was analyzed by RT-PCR. The total RNA of SK-N-SH cells was extracted using the RNeasy Mini Kit (Qiagen, Hilden, Germany). Cell cDNA was prepared with a PrimeScript RT-PCR Kit (Takara, Dalian, China) using random primers. Quantitative RT-PCR was performed with Taqman Universal PCR Master Mix (Applied Biosystems, Foster City, CA) in an ABI 7500 Real Time PCR System (Applied Biosystems, Foster City, CA). The FUS/TLS and 18S rRNA primer/probe sets (Applied Biosystems, Foster City, CA) were purchased from commercial sources. The relative quantification of FUS/TLS mRNA was analyzed using the manufacturer's software with 18S rRNA as the reference gene. To perform semiquantitative RT-PCR, the PCR products were electrophoresed on an agarose gel. Primer sequences are shown in Table 1.

### 2.13. Immunofluorescence Analysis

SK-N-SH cells stably expressing FUS/TLS R521C were seeded onto 4-well Lab-Tek II CC chamber slides (Nalge NUNC International, Rochester, NY). The cells were treated with or without PTE (200 *μ*g/mL) for 24 h, washed once with PBS, and fixed with 4% paraformaldehyde in PBS (5 min at RT). Following washing with PBS, the cells were permeabilized with 0.5% Triton X-100 in PBS for 10 min and blocked with PBS containing 1% BSA (Sigma-Aldrich, St. Louis, USA) with 0.05% Tween 20 for 30 min. The cells were incubated overnight at 4°C with mouse anti-V5 (1 : 1000) antibody, washed with PBS/0.05% Tween 20 (PBST), and incubated with biotinylated anti-mouse IgG antibody (1 : 200; Vector Laboratories, Burlingame, CA) and Alexa Fluor 594—strepavidin conjugate (1 : 100; Invitrogen, Carlsbad, CA) for 30 min. After washing with PBST, the coverslips were mounted on slides with Prolong Gold antifade reagent containing 4′,6′-diamidino-2-phenylindole (DAPI) (Invitrogen, Carlsbad, CA). The cells were observed using a confocal Zeiss LSM 510 microscope (Carl Zeiss Ltd., Hertfordshire, UK).

### 2.14. Statistical Analysis

All experiments were repeated at least three times. Data are expressed as the mean ± SEM. The statistical differences were evaluated using one-way analysis of variance (ANOVA) and Student's *t*-test.* P *values of less than 0.05 were considered significant.

## 3. Results

### 3.1. Purification and Identification of PTE-Associated Protein

**Figure 1:**
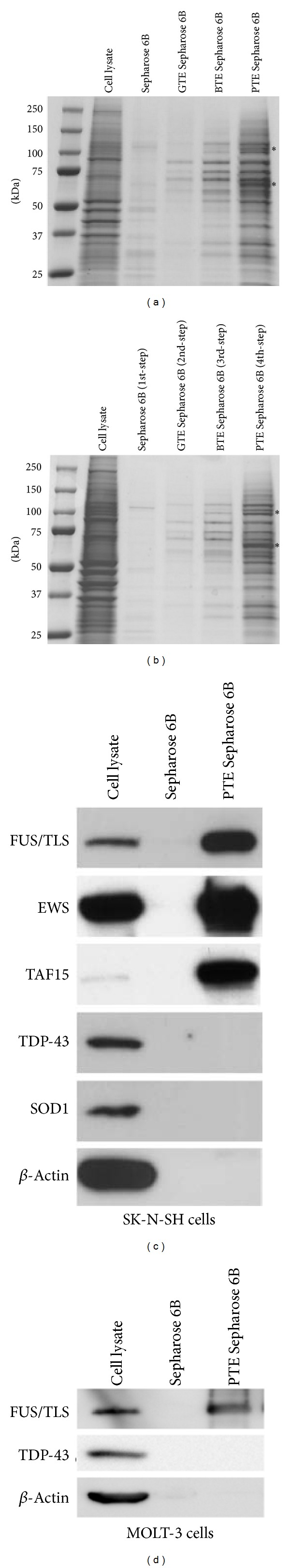
Purification and identification of the PTE associated protein. (a) Proteins derived from MOLT-3 cells were, respectively, loaded onto the four types of Sepharose 6B beads, and the proteins that were bound to the beads were analyzed on SDS-PAGE gels and stained with Coomassie brilliant blue. (b) A four-step purification procedure of PTE-associated proteins was performed. MOLT-3 cell lysates were sequentially loaded onto Sepharose 6B beads, GTE Sepharose 6B beads, and BTE Sepharose 6B beads, followed by PTE Sepharose 6B beads. The PTE-associated proteins were finally purified with PTE Sepharose 6B beads. The proteins, as indicated by the arrow, were removed from the gel and analyzed using MALDI-TOF MS. (c) SK-N-SH cell lysates and (d) MOLT-3 cell lysates were incubated with PTE Sepharose 6B or Sepharose 6B beads and analyzed by Western blot.

In order to isolate the PTE-associated protein, Sepharose 6B beads, GTE Sepharose 6B beads, BTE Sepharose 6B beads, and PTE Sepharose 6B beads were prepared. Proteins obtained from the MOLT-3 cells were loaded onto the four types of Sepharose 6B beads and mixed overnight at 4°C, and the proteins that bound to the four types of Sepharose 6B beads were then analyzed by SDS-PAGE. Two protein bands were detected (70 kDa and 130 kDa) that were bound to the PTE Sepharose 6B beads but not to GTE or BTE Sepharose 6B beads (Figure 1(a)). To separate the protein bands from each other, a four-step procedure for the purification of the PTE-associated proteins was performed. MOLT-3 cell lysates were sequentially loaded onto Sepharose 6B beads, GTE Sepharose 6B beads, BTE Sepharose 6B beads, followed by PTE Sepharose 6B beads. The proteins that bound to the beads were subjected to SDS-PAGE. The 70 kDa and 130 kDa protein bands were observed, which suggested that the 70 kDa and 130 kDa proteins were specific for associating with PTE (Figure 1(b)). To identify the 70 kDa protein and 130 kDa, the protein bands were excised from the gel and analyzed by MALDI-TOF MS. These mass spectrums were compared with protein databases, and the 70 KDa and 130 KDa proteins were identified as FUS/TLS (Table 2) and Heterogeneous nuclear ribonucleoprotein A1 (hnRNP A1) (data not shown), respectively.

### 3.2. Confirmation of FET Family Proteins as PTE Associated Proteins

Further evidence for the interaction between FUS/TLS protein and PTE was obtained by a pull-down assay using total proteins obtained from SK-N-SH cells, a human neuroblastoma cell line, Sepharose 6B beads, and PTE Sepharose 6B beads. The results showed that FUS/TLS bound to the PTE Sepharose 6B beads, but not control Sepharose 6B beads (Figure 1(c)). EWS and TAF15, which belong to FET family as well, also bound to the PTE Sepharose beads (Figure 1(c)). However, TDP-43, SOD1, and *β*-actin were not associated with the PTE Sepharose 6B beads (Figure 1(c)), suggesting that FET family proteins interacted specifically with PTE. The same results were observed with MOLT-3 cells (Figure 1(d)). Therefore, these results indicate that PTE interacts with FET family proteins.

### 3.3. Degradation of FET Family Proteins by PTE

**Figure 2:**
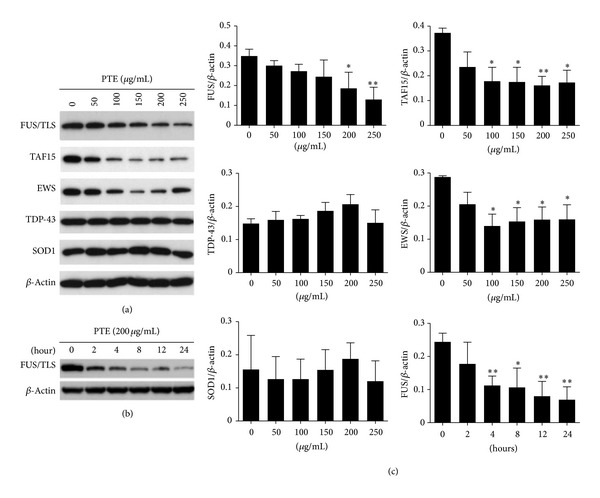
Decreased FET family protein expression by PTE in SK-N-SH cells. (a) Cells were treated with different concentrations of PTE for 24 h; the expression of FUS/TLS, EWS, TAF15, TDP-43, SOD1, and *β*-actin was detected by Western blot. (b) Cells were treated with 200 *μ*g/mL PTE for different time periods; Western blot was performed. (c) The Western blot results were quantified and statistical analysis was performed. Values are mean ± SEM of three independent experiments. *P < 0.05, **P < 0.01, and ***P < 0.001 compared with untreated control.

**Figure 3:**
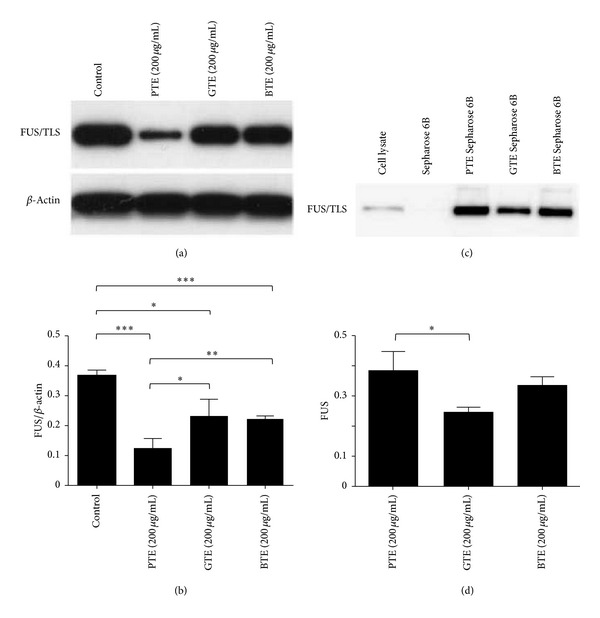
Effects of three different tea extracts on FUS/TLS in SK-N-SH cells. (a) Cells were treated with 200 *μ*g/mL PTE, GTE, or BTE for 24 h; the expression of FUS/TLS was detected by Western blot. (b) The Western blot results were quantified and statistical analysis was performed. Values are mean ± SEM of three independent experiments. *P < 0.05, **P < 0.01, and ***P < 0.001 compared with untreated control. (c) Cell lysates were incubated with PTE Sepharose 6B, GTE Sepharose 6B, BTE Sepharose 6B, or Sepharose 6B beads, and the levels of bound FUS/TLS were analyzed by Western blot. (d) The Western blot results were quantified and statistical analysis was performed. Values are mean ± SEM of three independent experiments. *P < 0.05.

To further investigate the physiological importance of the interaction between PTE and FET family proteins, we examined whether PTE affected the expression of FET family proteins. Different concentrations of PTE were added to SK-N-SH cells, and the samples were incubated for 24 h. The expression of FUS/TLS, EWS, TAF15 was significantly decreased after treatment with PTE (Figure 2(a)). In addition, the expression of FUS/TLS was reduced as early as 4 h after treatment with 200 *μ*g/mL PTE, and this occurred in a time-dependent manner (Figure 2(b)). In contrast, no reduction in the expression of TDP-43 protein or SOD1 protein was observed in cells that had been treated with PTE (Figure 2(a)). Similar results were observed in the case of MOLT-3 cells that had been treated with PTE (data not shown). Although GTE or BTE also decreased the expression of FUS/TLS, PTE induced the greatest decrease of FUS/TLS protein expression (Figures 3(a) and 3(b)). PTE bound to FUS/TLS stronger than GTE (Figures 3(c) and 3(d)).

**Figure 4:**
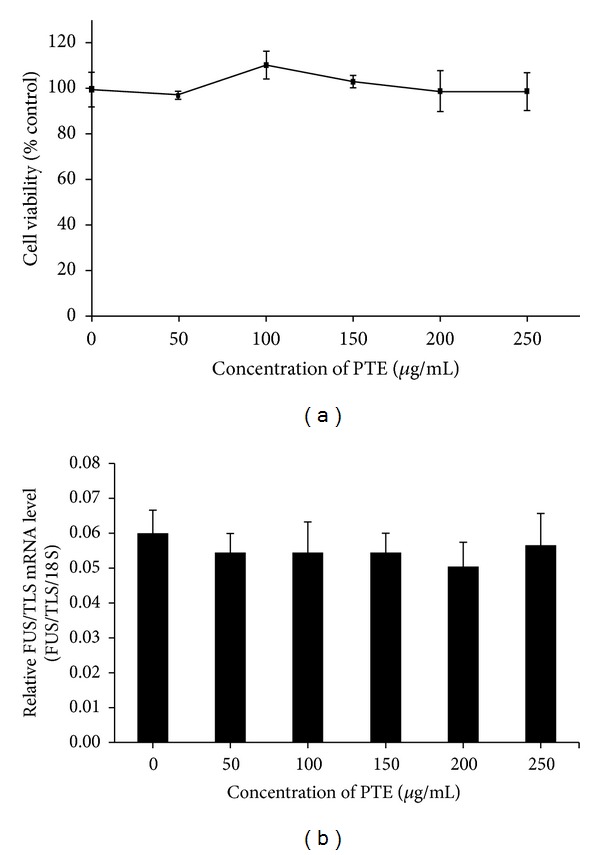
Effect of PTE on cell viability and FUS/TLS mRNA expression in SK-N-SH cells. Cells were treated with different concentrations of PTE for 24 h. (a) Cell viability was measured by trypan blue staining. Percentage viability was defined as the number of viable cells in treated versus untreated cells. (b) FUS/TLS mRNA expression was analyzed by quantitative RT-PCR. Values are mean ± SEM of three independent experiments. *P < 0.05, **P < 0.01, and ***P < 0.001 compared with untreated control.

To investigate whether PTE is toxic to SK-N-SH cells, we treated cells with various concentrations of PTE for 24 h and cell viability assays were performed. No significant difference in the levels of cell viability among cells treated with different concentrations of PTE was found (Figure 4(a)). These results indicate that PTE is not toxic to SK-N-SH cells. We next determined whether PTE led to the transcriptional inhibition of FUS/TLS. Quantitative RT-PCR was performed to detect FUS/TLS mRNA expression levels in SK-N-SH cells treated with PTE or not treated. No significant difference was found, compared with the control (Figure 4(b)). Similar results were observed in MOLT-3 cells (data not shown).

### 3.4. Lysosome-Dependent Degradation of FUS/TLS by PTE

**Figure 5:**
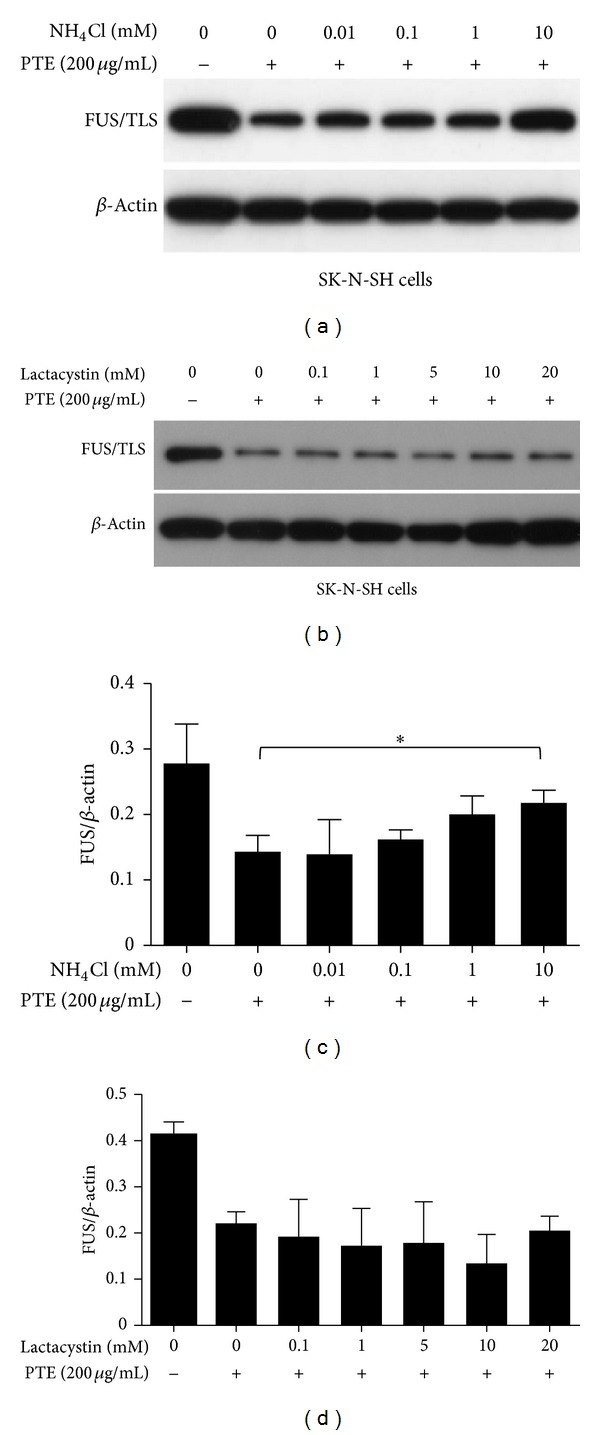
Lysosome-dependent degradation of FUS/TLS by PTE. SK-N-SH cells were incubated with or without inhibitor for 1 h, (a) ammonium chloride (NH_4_Cl), a lysosome inhibitor, or (b) lactacystin, a proteasome inhibitor, followed by treatment with PTE (200 *μ*g/mL) for 24 h. Cell lysates were detected by Western blot with anti-FUS/TLS. *β*-actin was used as the loading control. (c) (d) The Western blot results were quantified and statistical analysis was performed. Values are mean ± SEM of three independent experiments. *P < 0.05, **P < 0.01, and ***P < 0.001 compared with treated with PTE only.

The lysosomal system and the ubiquitin-proteasome system are responsible for the majority of intracellular protein degradation. To investigate the mechanism of FUS/TLS degradation, we assessed the effects of proteasome and lysosome inhibitors on PTE-induced FUS/TLS degradation. SK-N-SH cells were pretreated with lysosome or proteasome inhibitors for 1 h prior to PTE addition. FUS/TLS expression levels were assessed by Western blot. Pretreatment with NH_4_Cl prevented the PTE-induced FUS/TLS protein degradation (Figure 5(a)). However, Lactacystin did not affect PTE-induced FUS/TLS protein degradation in SH-N-SH cells (Figure 5(b)). These results indicate that PTE induces FUS/TLS protein degradation via the lysosome-dependent pathway.

### 3.5. Degradation of the Mutant FUS/TLS Protein by PTE

**Figure 6:**
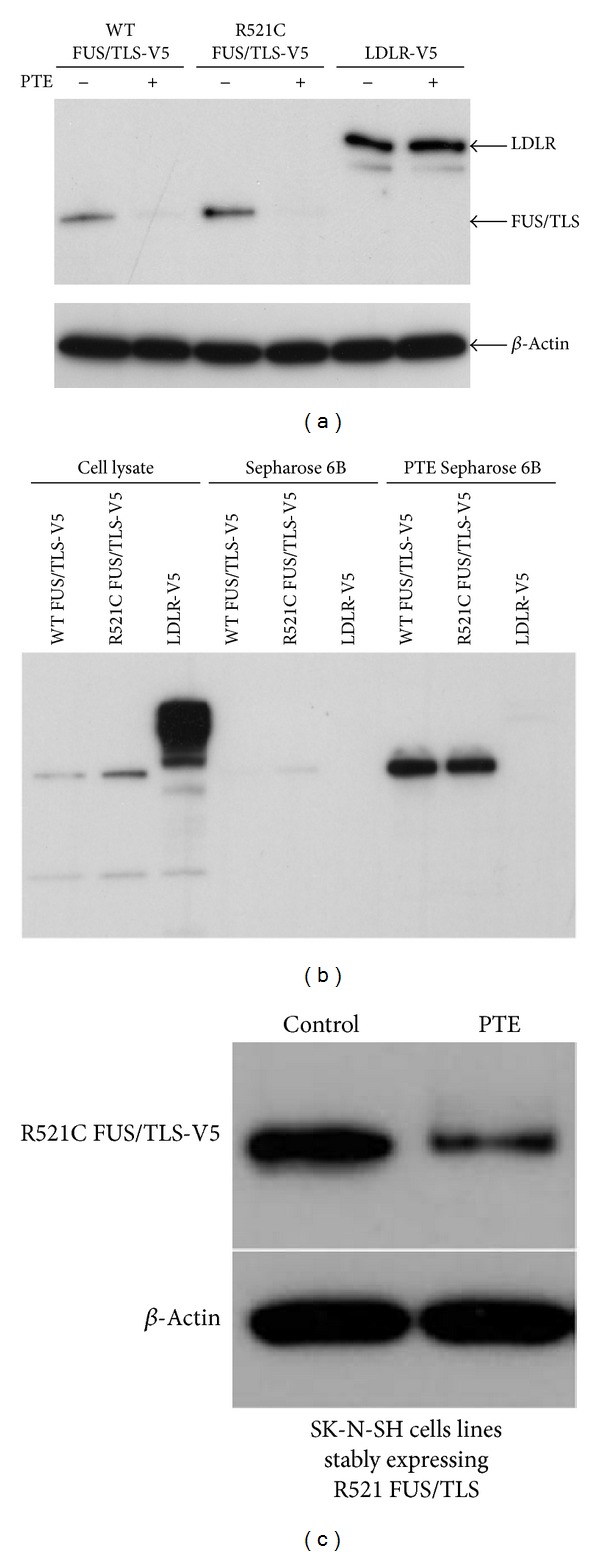
Degradation of R521C FUS/TLS protein by PTE. (a) SK-N-SH cells were transfected with V5-tagged wild-type FUS/TLS or R521C FUS/TLS or LDLR. Transfected cells were treated with or without PTE (200 *μ*g/mL) for 24 h. Cell lysates were detected by Western blot with anti-V5 antibody. (b) HEK 293T cells were transfected with V5-tagged wild-type FUS/TLS, R521C FUS/TLS, or LDLR. Protein extracts from transfected 293T cells were incubated with PTE Sepharose 6B or Sepharose 6B beads. Proteins that were bound to the beads were analyzed by Western blot with anti-V5 antibody. (c) SK-N-SH cells stably expressing R521C FUS/TLS were treated with or without PTE (200 *μ*g/mL) for 24 h. Cell lysates were detected by Western blot with anti-V5 antibody. *β*-actin was used as the loading control.

**Figure 7:**
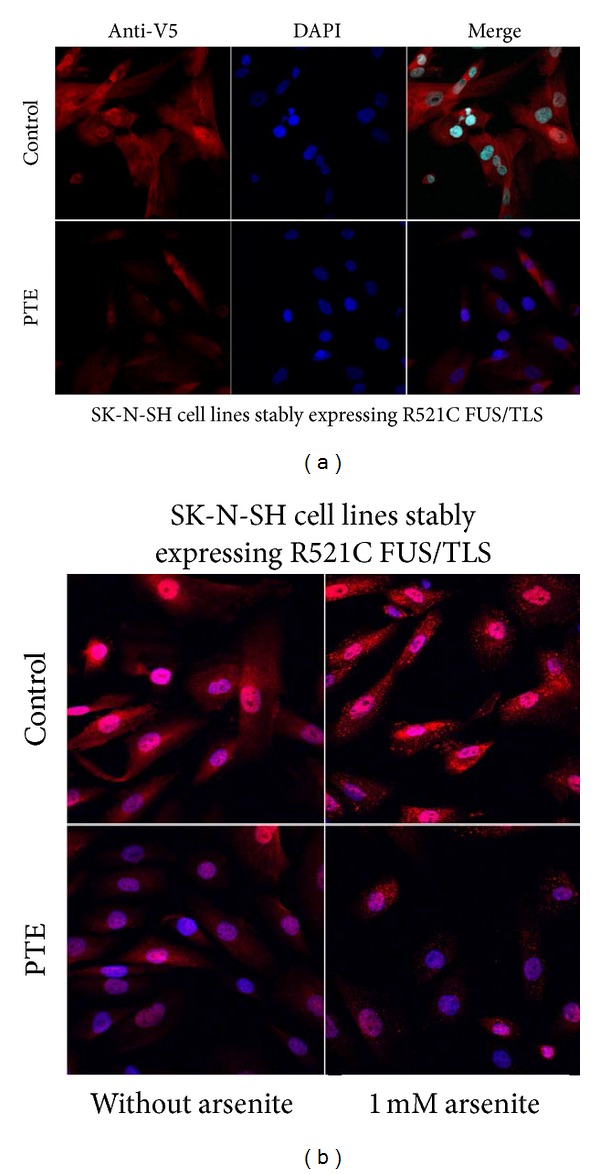
Reduction in FUS-positive cytoplasmic granules by PTE under stress conditions. (a) SK-N-SH cells stably expressing R521C FUS/TLS were treated with or without PTE (200 *μ*g/mL) for 24 h. (b) SK-N-SH cells stably expressing R521C FUS/TLS were treated with or without PTE (200 *μ*g/mL) for 24 h, following treatment with 1 mM arsenite. Cells were subsequently fixed and analyzed by immunofluorescence microscopy using anti-V5 antibody and DAPI.

We assessed whether PTE induces the degradation of mutant FUS/TLS proteins. The R521C FUS/TLS mutation is the most common form of mutation, and it is characterized by an early onset and rapid progress of ALS symptoms. We transfected the R521C mutant gene into SK-N-SH cells and treated transfected cells with or without PTE. PTE induced the degradation of R521C FUS/TLS proteins. In contrast, PTE had no effect on the level of LDLR protein in transfected cells (Figure 6(a)). The evidence for an interaction between R521C FUS/TLS and PTE was demonstrated by a pull-down assay using Sepharose 6B beads and PTE Sepharose 6B beads (Figure 6(b)). In order to further investigate the effect of PTE on R521C FUS/TLS proteins, SK-N-SH cells that stably expressed R521C FUS/TLS were established. In the SK-N-SH cells stably expressing R521C FUS/TLS, R521C FUS/TLS proteins had increased cytoplasmic location (Figure 7(a)). PTE induced the degradation of R521C FUS/TLS proteins (Figure 5(a)) and decreased the expression of the cytoplasmic R521C FUS/TLS proteins (Figure 7(a)).

### 3.6. Reduction of FUS-Positive Cytoplasmic Granules by PTE under Stress Conditions

FUS/TLS with ALS-causing mutations is incorporated into cytoplasmic stress granules under stress conditions and is important in the pathogenesis of ALS [24–27]. Sodium arsenite is commonly used to induce oxidative stress in* in vitro *models. To assess whether PTE affects the incorporation of mutant FUS/TLS into stress granules, we treated SK-N-SH cell lines stably expressing R521C FUS/TLS mutation (V5 tagged) with PTE (200 *μ*g/mL) for 24 h and with 1 mM sodium arsenite for 1 hour, which increases intracellular ROS [28] and induces stress granules formation [29]. Cells were immunostained with anti-V5 (red, FUS) and DAPI (blue, nuclear marker) and observed under confocal microscopy. R521C FUS/TLS was incorporated into cytoplasmic stress granules under stress condition. PTE significantly reduced the number of FUS-positive cytoplasmic granules following sodium arsenite treatment (Figure 7(b)).

## 4. Discussion

The findings reported here show that PTE inhibits the expression of FET family proteins, but it has no effect on FUS/TLS mRNA levels in SK-N-SH cells, which suggests that the inhibition occurs via a posttranscriptional mechanism. In addition, most of the cells survived after PTE treatment. This result indicates that the decrease in the level of FUS/TLS protein was not the result of cell death. Furthermore, the data clearly show that PTE interacts with FET family proteins. TDP-43 and SOD-1 failed to interact with PTE, and their expressions were not inhibited by PTE. PTE induced a decrease in FUS/TLS protein levels in SH-N-SH cells, which was rescued by a lysosome inhibitor. Importantly, PTE also induced the degradation of R521 FUS/TLS protein. These results imply that the interaction between PTE and FUS/TLS leads to the degradation of FUS/TLS and its mutant protein.

Tea is one of the most popular and widely consumed beverages in the world. Unlike green tea, pu-erh tea contains a number of compounds that are produced as the result of the preparation process. Human epidemiological and animal research experiments suggest that the pharmacological benefits of tea drinking may help protect the brain as we age. There is a growing body of evidence to indicate that polyphenol compounds have the ability to convert large, mature *α*-synuclein, and amyloid-*β* fibrils into smaller, amorphous protein aggregates that are nontoxic to mammalian cells [30–32]. Epigallocatechin gallate (EGCG) significantly reduces the aggregation and cytotoxicity of the Huntington protein containing polyglutamine (polyQ) in a yeast model of Huntington's disease [33]. It should also be noted that tea drinking has been shown to exert neuroprotective activities in a wide array of cellular and animal models of neurological disorders [34]. In this study, we prepared three different tea extracts Sepharose beads to screen the proteins strongly associated with PTE. FUS/TLS was identified as a PTE-associated protein. Although FUS/TLS also could bind to GTE and BTE, PTE did have the greatest capacity to bind to FUS/TLS among those three different tea extracts. Perhaps this may account for the apparent absence of FUS/TLS in the proteins that bind to GTE or BTE Sepharose 6B beads in Coomassie brilliant staining. EGCG is the most abundant catechin in tea. EGCG is found mainly in green tea but, in smaller quantities, in black tea and Pu-erh Tea. We were not able to observe the degradation of FUS/TLS after treatment with EGCG (data not shown).

Protein aggregation in neurons is associated with a number of neurodegenerative disorders. Exactly how FET and TDP-43, either the mutant or wild-type form, contribute to these diseases remains unclear. Two main potential mechanisms have been proposed. First, since FET and TDP-43 are both RNA-binding proteins, these proteins are normally located in the nucleus and are involved in RNA processing. Therefore, the aggregation of proteins in the cytoplasm inhibits their physiological function. Second, the cytoplasmic aggregations formed from these proteins are toxic to cells. ALS-linked FUS mutations are mostly localized to the cytoplasm. Following stress, FUS/TLS is incorporated into stress granules, thereby initiating the aggregation and sequestration of wild-type FUS/TLS [35]. We have also shown that cytoplasmic R521C FUS is incorporated into stress granules following cellular stress in SK-N-SH cell lines stably expressing R521C FUS/TLS. The results of the current study raise questions concerning the physiological significance of the FET family proteins degradation induced by PTE. PTE may prevent protein aggregation and enable cells to function normally with normal levels of FET family proteins. There are currently no FET family protein-targeted therapies available for the treatment of ALS. The decreased levels of a toxic FET family proteins or mutant FET family proteins might be efficacious for FET family protein-associated ALS. Further studies are required to assess whether the PTE-induced reduction in FET family proteins is also observed in* in vivo* FET family proteins proteinopathies models.

## 5. Conclusion

The findings of this study revealed that PTE interacted with FET family proteins but not with TDP-43 or SOD1 protein. PTE induced the degradation of FET family proteins but had no effect on TDP-43 or SOD1. Additionally, PTE induced the degradation of R521C FUS/TLS protein. PTE significantly decreased the expression of cytoplasmic R521C FUS/TLS protein and the number of FUS-positive cytoplasmic granules under stress conditions. These findings contribute to our understanding of the molecular mechanism underlying the antineurodegenerative effects of PTE and suggest that pu-erh tea maybe helpful in preventing the onset of FET family protein-associated neurodegenerative diseases and in delaying the progression of these types of diseases through inhibiting the cytoplasmic aggregation of FET family proteins.

## Figures and Tables

**Table 1:** Primer sequences.

Primer	Sequence (5′ to 3′)
*FUS/TLS* WT forward	ACCATGGCCTCAAACGATTATACCCAAC
*FUS/TLS* WT reverse	ATACGGCCTCTCCCTGCGATCCTGTCTG
*FUS/TLS* R521C forward	ACCATGGCCTCAAACGATTATACCCAAC
*FUS/TLS* R521C reverse	ATACGGCCTCTCCCTGCAATCCTG
*LDLR* forward	ACCATGGGGCCCTGGGGCTGGAAATTG
*LDLR* reverse	CGCCACGTCATCCTCCAGACTGAC
*GADPH* forward	GAAGGTGAAGGTCGGAGTC
*GADPH* reverse	GAAGATGGTGATGGGATTTC

**Table 2:** Identification of the PTE binding protein based on peptide fragments produced by MAILDI-TOF MS.

Peptide	Start–end	Observed	Mr (expt.)	Mr (calc.)	Delta *M*
K.KTGQPMINLYTDR.E	90–102	1536.7841	1535.7768	1535.7766	0.0002
K.KTGQPMINLYTDR.E + oxidation (M)	90–102	1552.7707	1551.7635	1551.7715	−0.0080
K.TGQPMINLYTDR.E	90–102	1408.7088	1407.7015	1407.6816	0.0199
K.TGQPMINLYTDRETGK.L	91–106	1823.8919	1822.8847	1822.8883	−0.0037
K.LKGEATVSFDDPPSAK.A	107–122	1661.8517	1660.8444	1660.8308	0.0136
K.AAIDWFDGKEFSGNPIK.V	123–139	1894.9324	1893.9251	1893.9261	−0.0010
K.CPNPTCENMNFSWP.N	202–215	1698.6680	1697.6608	1697.6749	−0.0141
K.APKPDGPGGGPGGSHMGGNYGDDR.R	223–246	2252.9478	2251.9406	2251.9665	−0.0259
NCBI database		GI:62087384			
